# The impact of lockdown during the COVID-19 pandemic on osteoporotic fragility fractures: an observational study

**DOI:** 10.1007/s11657-020-00825-1

**Published:** 2020-10-07

**Authors:** Giulia Ogliari, Eleanor Lunt, Terence Ong, Lindsey Marshall, Opinder Sahota

**Affiliations:** 1 Department of Health Care for Older People (HCOP), Queen’s Medical Centre, Nottingham University Hospitals NHS Trust, Derby Road, Nottingham, Nottinghamshire, NG7 2UH UK; 2 Faculty of Medicine, University of Malaya, Kuala Lumpur, Malaysia; 3 Department of Trauma & Orthopaedics, Queen’s Medical Centre, Nottingham University Hospitals NHS Trust, Nottingham, UK; 4 University of Nottingham , Nottingham, UK

**Keywords:** COVID-19, Older adults, Fragility fracture, Hip fracture, Secondary healthcare utilisation

## Abstract

***Summary*:**

We investigated whether osteoporotic fractures declined during lockdown, among adults aged 50 years and older. We showed that fewer outpatients attended the Fracture Clinic, for non-hip fractures, during lockdown; in contrast, no change in admissions for hip fractures was observed. This could be due to fewer outdoors falls, during lockdown.

**Purpose:**

Many countries implemented a lockdown to control the spread of the COVID-19 pandemic. We explored whether outpatient attendances to the Fracture Clinic for non-hip fragility fracture and inpatient admissions for hip fracture declined during lockdown, among adults aged 50 years and older, in a large secondary care hospital.

**Methods:**

In our observational study, we analysed the records of 6681 outpatients attending the Fracture Clinic, for non-hip fragility fractures, and those of 1752 inpatients, admitted for hip fracture, during the time frames of interest. These were weeks 1st to 12th in 2020 (“prior to lockdown”), weeks 13th to 19th in 2020 (“lockdown”) and corresponding periods over 2015 to 2019. We tested for differences in mean numbers (standard deviation (SD)) of outpatients and inpatients, respectively, per week, during the time frames of interest, across the years.

**Results:**

Prior to lockdown, in 2020, 63.1 (SD 12.6) outpatients per week attended the Fracture Clinic, similar to previous years (*p* value 0.338). During lockdown, 26.0 (SD 7.3) outpatients per week attended the Fracture Clinic, fewer than previous years (*p* value < 0.001); similar findings were observed in both sexes and age groups (all *p* values < 0.001). During lockdown, 16.1 (SD 5.6) inpatients per week were admitted for hip fracture, similar to previous years (*p* value 0.776).

**Conclusion:**

During lockdown, fewer outpatients attended the Fracture Clinic, for non-hip fragility fractures, while no change in inpatient admissions for hip fracture was observed. This could reflect fewer non-hip fractures and may inform allocation of resources during pandemic.

**Electronic supplementary material:**

The online version of this article (10.1007/s11657-020-00825-1) contains supplementary material, which is available to authorized users.

## Introduction

In December 2019, a novel coronavirus, the severe acute respiratory syndrome coronavirus 2 (SARS-CoV-2), emerged and caused a pandemic [1]. COVID-19, the disease caused by SARS-CoV-2, is characterised by fever, respiratory symptoms as well as fatigue, myalgia, dizziness and delirium [1, 2]. Under guidance from the World Health Organisation (WHO), many countries implemented a lockdown to control the spread of the disease. In the UK, emergency legislation restricted the movement of people from where they lived, except that for basic necessities including medical care, an initial strict lockdown period ran in the UK from Monday 23rd March 2020 to Tuesday 12th May 2020, with a subsequent relaxation of the rules [3].

It is unknown whether the restriction of outdoor movements imposed by the lockdown may affect the incidence of osteoporotic fragility fractures, by potentially reducing the number of outdoor falls and subsequent fractures, among older adults [4–7]. This effect could be particularly pronounced on non-hip fragility fractures (including forearm, upper arm, ankle, foot and others), compared with hip fractures. While hip fractures more frequently occur indoors and affect frail older adults, several types of non-hip fractures occur outdoors and affect more physically active older adults [7–11]. On the other hand, COVID-19, and its associated symptoms of fatigue, dizziness and delirium, could contribute to falls and fractures among older people.

Osteoporotic fragility fractures are a major public health and healthcare issue worldwide, as they are associated with high healthcare costs, morbidity and mortality [12]. Recent literature has suggested that non-hip fractures as a whole may be more common and costly than hip fractures [13]. Seasonality has been shown for hip and non-hip fragility fractures, with highest incidence during winter months, in many countries [9, 14–20]. While hip fractures generally require hospital admission, non-hip fractures are generally managed through outpatient Fracture Clinics. We expected that fewer older outpatients may attend the Fracture Clinic for non-hip fractures, during lockdown. If proven, this could be relevant for allocation of limited healthcare resources, during a pandemic.

The aim of this study was to investigate whether lockdown may be associated with a reduction in the number of older outpatients attending the Fracture Clinic, for any type of acute non-hip fragility fractures and reduction in inpatient hospital admissions for acute hip fracture, in a large secondary care hospital, compared with corresponding periods, across previous years. In addition, we describe the demographic and clinical characteristics of older outpatients, attending the Fracture Clinic, during lockdown.

## Materials and methods

### Study design

The Queen’s Medical Centre (QMC), Nottingham University Hospitals NHS Trust, is one of the largest university hospitals in the UK [21, 22], with a catchment population of 750,000. It provides universal health coverage, free of charge to adults with suspected acute fractures, as part of the National Health Service (NHS). Those adults with a suspected acute fracture are assessed at the QMC’s Major Trauma Centre, Emergency and Accident Department; those adults with hip fracture are admitted to hospital for orthopaedic surgery while those adults with non-hip fractures that do not require hospitalisation are referred to the outpatient Fracture Clinic and seen there within one to three days; the need for repeated radiographs and resetting of plaster casts warrant physical attendance to the Fracture Clinic. The Fracture Liaison Service systematically identifies those adults aged 50 years and older with low trauma fragility fractures, including both those attending the Fracture Clinic and those hospitalised for an acute hip fracture. The Fracture Liaison Services are well established in the UK [23–26]; the Nottingham Fracture Liaison Service of QMC has systematically collected routine clinical data of all patients with low-trauma, fragility fractures, in a structured database, since 2008 [27]. We undertook a data analysis of the Nottingham Fracture Liaison Service Database of outpatients aged 50 years and older, with a fragility fracture attending the Fracture Clinic and those of inpatients aged 50 years and older, hospitalised for an acute hip fracture.

### Time frames

These time frames of interest were selected: weeks 1st to 12th, in 2020 (“prior to lockdown”); weeks 13th to 19th, in 2020 (“during lockdown”, corresponding to the full seven weeks of strict lockdown in the UK) and the corresponding periods over the previous five years (2015 to 2019). The weeks were numbered according to the UK calendar rules, where Monday begins the week (Supplementary Table 1) [28]. In further analyses, we divided the “weeks 1st to 12th” period into three 4-week periods and the “weeks 13th to 19th” period into two parts—weeks 13th to 16th and weeks 17th to 19th; this was done taking into account the seasonality of fragility fractures and a possible adjustment phase during lockdown. During lockdown, the capacity of the Fracture Clinic and inpatient beds allocated for hip fracture care remained the same, but all elective (non-emergency) orthopaedic activity was reduced.

### Study participants

**Fig. 1:**
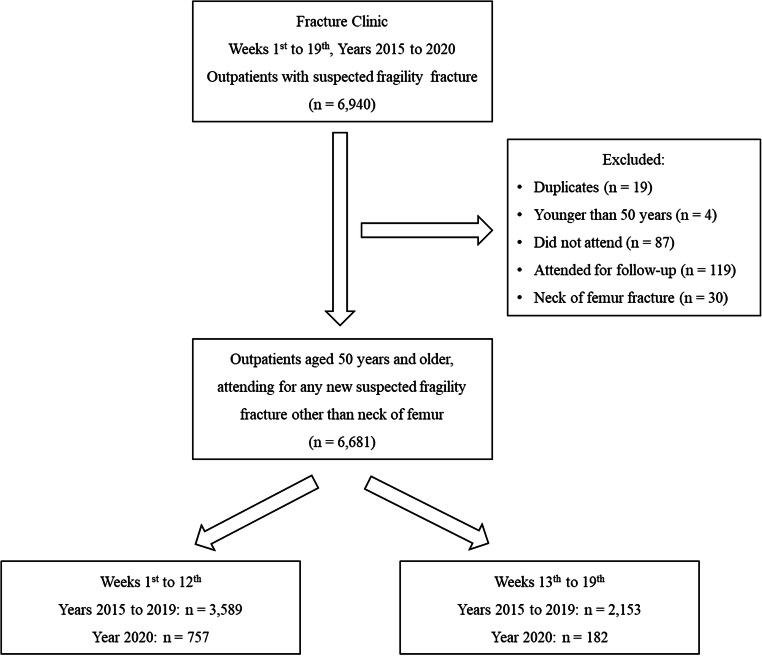
Flowchart of study design

We identified the database records for outpatients, who attended the Fracture Clinic, following a new suspected fragility fracture, and for inpatients who were admitted to hospital, following an acute hip fracture, during the time frames of interest. In the outpatient cohort, a total of 6940 outpatients were referred to the Fracture Clinic, during the time frames of interest (weeks 1st to 19th, years 2015 to 2020). After removal of duplicates (*n* = 19) and exclusion of those younger than 50 years (*n* = 4), those who did not attend (*n* = 87) or attended for follow-up of a previously identified fracture (*n* = 119) and those who were diagnosed with a hip fracture (*n* = 30), we included in our study 6681 outpatients aged 50 years or older, who attended the Fracture Clinic for a new suspected fragility fracture, other than hip (Fig. 1, flowchart of study design).

In the inpatient cohort, a total of 3242 inpatients were admitted to the Trauma and Orthopaedics wards during the time frames of interest (weeks 1st to 19th, years 2015 to 2020). After excluding those with no hip fracture or with pathological or periprosthetic or not classed as low trauma or fragility hip fracture, and those younger than 50 years, we included in our study 1752 inpatients aged 50 years or older, admitted to hospital for an acute hip fragility fracture.

### Demographic and clinical characteristics

Sociodemographic (age, sex), administrative (date of visit) and clinical (type of fracture) data were retrieved from the outpatients’ records (we had no missing data for these variables). Types of non-hip fractures were categorised as follows: radius, ulna or humerus; clavicle, scapula or shoulder; ankle or foot; tibia, fibula, patella or femur (excluding neck of femur); other types of fracture (including metacarpal, hand or finger(s), …); no fracture (including tendon rupture, dislocation, inversion, avulsion and no definite fracture). We also categorised the outpatients into four age/sex categories: women aged 65 years or older, women aged 50 to 64 years, men aged 65 years or older, men aged 50 to 64 years. Data on age, sex and date of admission were retrieved for the inpatients with acute hip fracture (we had no missing data for these variables).

### Statistical analysis

We used SPSS version 17 (SPSS Inc., Chicago, Ill., USA) for all the analyses.

We calculated the mean (standard deviation (SD)) number of all outpatients attending the Fracture Clinic, during the time frames of interest, across the years. We tested for differences in mean number of outpatients between the years, by using analysis of variance (ANOVA) and post-hoc Tukey’s test. We repeated these analyses after stratifying by sex and age (patients aged 50 to 64 years and aged 65 years and older), respectively. Furthermore, we repeated all these analyses by including only those outpatients with a new confirmed fragility fracture.

Likewise, we calculated the mean (SD) number of inpatients, who were admitted to hospital, following an acute hip fracture, during the time frames of interest, and similarly tested for differences between the years.

We plotted the age distribution of the outpatients; as it was not normally distributed, we reported the median age (interquartiles (IQs)) and tested for differences between the years by non-parametric Kruskal-Wallis test. We reported the other demographic and clinical characteristics of the outpatients as frequency (percentage) and tested for differences between the years by using chi-square test. Similar analyses were performed in the inpatient cohort.

### Institutional Review Board approval

No Institutional Review Board approval was needed for this study, which is based on local data that are routinely collected as part of the national “Falls and Fragility Fracture Audit Programme” [29].

## Results

**Fig. 2:**
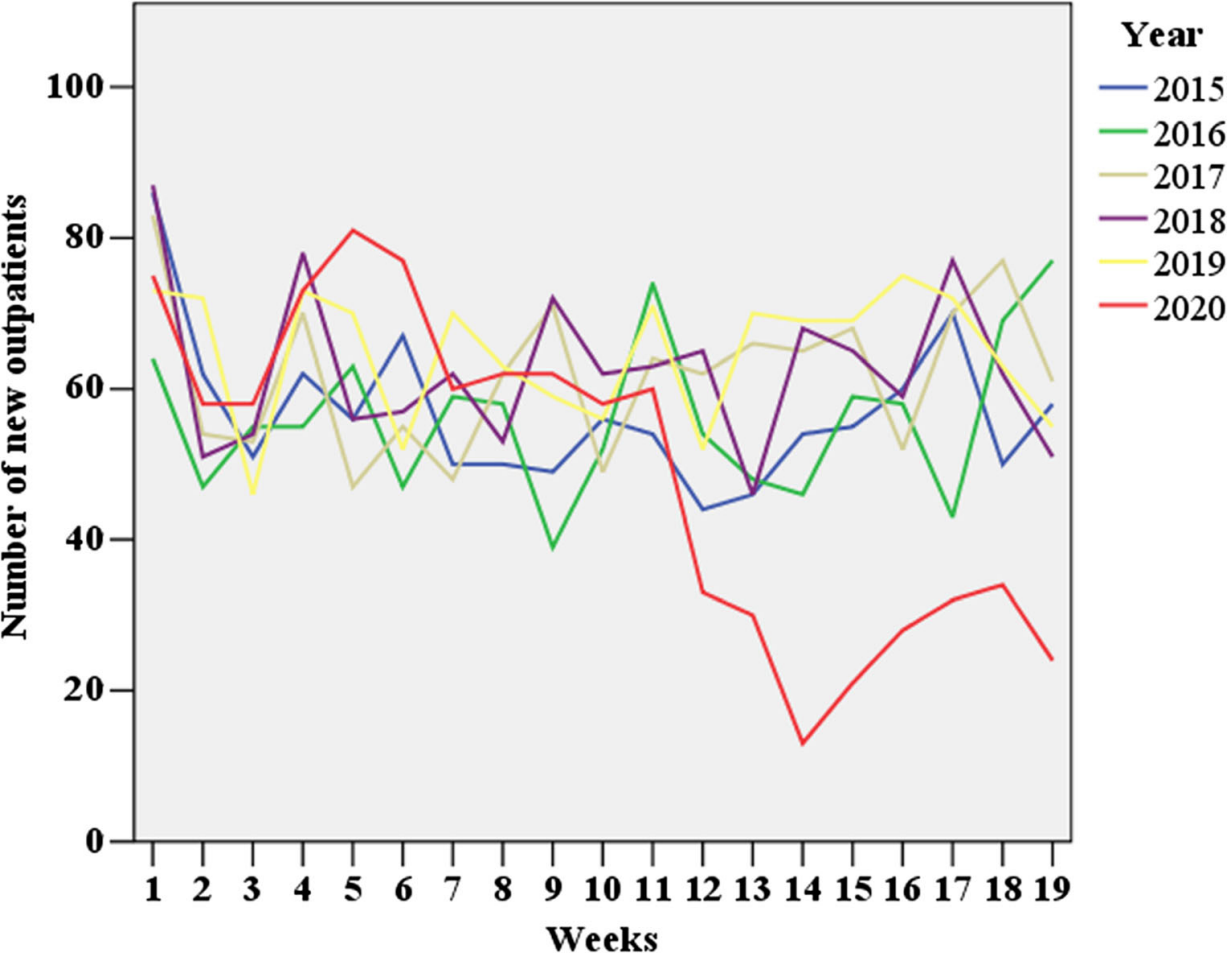
New outpatients to the Fracture Clinic in the first 19 weeks of the year, across the years 2015 to 2020

A total of 182 outpatients aged 50 years and older attended the Fracture Clinic during lockdown, in 2020. This figure was lower than those observed in corresponding periods, across the previous five years (2015 - 2019) (Fig. 2 and Table 1). Similar findings were observed when stratifying by age and sex, respectively (Table 1, Supplemental Figure 1 and 2).

**Table 1:** New outpatients attending the Fracture Clinic prior to lockdown and during lockdown in 2020 and in corresponding periods in the years 2015 to 2019

	2015	2016	2017	2018	2019	2020	*p* value
Weeks 1st to 12th (prior to lockdown)							
All outpatients (*n*)	687	667	718	760	757	757	
All outpatients (mean *n* (SD), per week)	57.3 (11.2)	55.6 (9.2)	59.8 (10.9)	63.3 (10.9)	63.1 (9.7)	63.1 (12.6)	0.338
Women (*n*)	461	447	494	526	523	498	
Women (mean *n* (SD), per week)	38.4 (7.1)	37.3 (6.8)	41.2 (8.7)	43.8 (10.9)	43.6 (7.2)	41.5 (9.2)	0.318
Men (*n*)	226	220	224	234	234	259	
Men (mean *n* (SD), per week)	18.8 (5.3)	18.3 (5.2)	18.7 (4.6)	19.5 (3.1)	19.5 (3.7)	21.6 (5.3)	0.576
Aged 50 to 64 years (*n*)	330	317	339	376	340	373	
Aged 50 to 64 years (mean *n* (SD), per week)	27.5 (7.5)	26.4 (6.4)	28.3 (6.9)	31.3 (7.1)	28.3 (6.2)	31.1 (8.7)	0.480
Aged ≥65 years (*n*)	357	350	379	384	417	384	
Aged ≥65 years (mean *n* (SD), per week)	29.8 (5.8)	29.2 (5.9)	31.6 (5.9)	32.0 (5.8)	34.8 (7.0)	32.0 (6.8)	0.315
Weeks 13th to 19th (during lockdown)							
All outpatients (*n*)	393	400	459	428	473	182	
All outpatients (mean *n* (SD), per week)	56.1 (7.7)	57.1 (12.6)	65.6 (7.8)	61.1 (10.4)	67.6 (6.6)	26.0 (7.3)*	< 0.001
Women (*n*)	281	272	308	301	318	118	
Women (mean *n* (SD), per week)	40.1 (5.1)	38.9 (8.9)	44.0 (4.7)	43.0 (7.4)	45.4 (7.0)	16.9 (4.6)*	< 0.001
Men (*n*)	112	128	151	127	155	64	
Men (mean *n* (SD), per week)	16.0 (2.6)	18.3 (4.7)	21.6 (5.1)	18.1 (6.6)	22.1 (4.1)	9.1 (3.2)^#^	< 0.001
Aged 50 to 64 years (*n*)	181	201	226	193	208	96	
Aged 50 to 64 years (mean *n* (SD), per week)	25.9 (6.9)	28.7 (9.3)	32.3 (4.9)	27.6 (3.9)	29.7 (6.1)	13.7 (3.8)*	< 0.001
Aged ≥65 years (*n*)	212	199	233	235	265	86	
Aged ≥65 years (mean *n* (SD), per week)	30.3 (6.8)	28.4 (6.0)	33.3 (4.6)	33.6 (7.8)	37.9 (5.2)	12.3 (4.2)*	< 0.001

Trend analyses for mean numbers of outpatients per week were performed with analysis of variance (ANOVA). Post hoc Tukey’s test: *significant difference between the year 2020 and all the other years; ^**#**^significant difference between the year 2020 and the years 2016, 2017, 2018 and 2019 (all *p* values < 0.05)

*Abbreviation*: *SD*, standard deviation

Prior to lockdown, in 2020, the mean number of new outpatients per week did not differ compared with those in corresponding periods, across 2015 to 2019 (Table 1). Prior to lockdown, in 2020, a mean number of 63.1 (SD 12.6) outpatients per week attended the Fracture Clinic, while during lockdown, in 2020, this number dropped to 26.0 (SD 7.3) outpatients. The mean number of all outpatients during lockdown, in 2020, was significantly lower than those in previous years (*p* value < 0.001); a similar reduction was observed also when stratifying by sex or age (all *p* values < 0.001, Table 1 and Supplemental Figure 1 and 2).

Similar findings were observed when dividing the prior to lockdown and lockdown period into shorter 4-week periods and 13th to 16th week and 17th to 19th week periods, respectively (Supplemental Table 2).

We performed sensitivity analyses by including only those outpatients with a new confirmed fragility fracture (*n* = 4796). Results did not materially change (Supplemental Table 3). Fewer outpatients attended the Fracture Clinic for a new confirmed fracture, during lockdown (mean number per week 18.0 (SD 4.1)), compared with previous years (mean number per week 44.1 (SD 7.2)) (*p* value < 0.001).

During lockdown, most outpatients attending the Fracture Clinic were female (*n* = 118, 64.8%), with a median age of 63 years (IQs 56; 73) (Table 2). The most common type of fractures were those of the radius, ulna and humerus combined (*n* = 73, 40.1%), followed by those of the ankle or foot (*n* = 23, 12.6%). During lockdown, outpatients were younger compared with previous years (*p* value 0.016, Table 2). The distribution of types of fractures during lockdown differed compared with previous years; in particular, the proportion of fractures of the ankle or foot during lockdown was lower compared with previous years (*p* value 0.003, Table 2).

**Table 2:** Characteristics of the new outpatients attending the Fracture Clinic during lockdown in 2020 and in corresponding periods in the years 2015 to 2019 (weeks 13th to 19th)

	2015 (*n* = 393)	2016 (*n* = 400)	2017 (*n* = 459)	2018 (*n* = 428)	2019 (*n* = 473)	2020 (*n* = 182)	*p* value
Women (*n* (%))	281 (71.5)	272 (68.0)	308 (67.1)	301 (70.3)	318 (67.2)	118 (64.8)	0.515
Age (years) (median (IQ))	66 (57; 77)	64 (56; 77)	65 (56; 74)	67 (57; 77)	67 (58; 78)	63 (56; 73)	0.016
Aged ≥85 years (*n* (%))	48 (12.2)	45 (11.3)	33 (7.2)	45 (10.5)	53 (11.2)	15 (8.2)	0.153
Age/sex category (*n* (%)):							0.169
Women ≥65 years	169 (43.0)	147 (36.8)	169 (36.8)	181 (42.3)	181 (38.3)	64 (35.2)	
Women aged 50 to 64 years	112 (28.5)	125 (31.3)	139 (30.3)	120 (28.0)	137 (29.0)	54 (29.7)	
Men ≥65 years	43 (10.9)	52 (13.0)	64 (13.9)	54 (12.6)	84 (17.8)	22 (12.1)	
Men aged 50 to 64 65 years	69 (17.6)	76 (19.0)	87 (19.0)	73 (17.1)	71 (15.0)	42 (23.1)	
Type of fracture (*n* (%)):							0.003
Radius, ulna or humerus	178 (45.3)	171 (42.8)	178 (38.8)	182 (42.5)	191 (40.4)	73 (40.1)	
Clavicle, scapula or shoulder	22 (5.6)	12 (3.0)	29 (6.3)	16 (3.7)	23 (4.9)	8 (4.4)	
Ankle or foot	78 (19.8)	81 (20.3)	101 (22.0)	79 (18.5)	82 (17.3)	23 (12.6)	
Tibia, fibula, patella or femur	9 (2.3)	11 (2.8)	19 (4.1)	31 (7.2)	19 (4.0)	14 (7.7)	
Other (metacarpal, hand or finger(s), …)	3 (0.8)	6 (1.5)	8 (1.7)	7 (1.6)	8 (1.7)	8 (4.4)	
No fracture	103 (26.2)	119 (29.8)	124 (27.0)	113 (26.4)	150 (31.7)	56 (30.8)	

*p* values are calculated using chi-square test for categorical variables and Kruskal-Wallis test for differences in median age

*Abbreviations*: *n*, number; *IQ*, interquartiles

In contrast, the mean number of new inpatient admissions for acute hip fracture per week remained unchanged, during lockdown, in 2020, compared with corresponding periods, across the previous years (Supplemental Table 4). The clinical characteristics of these inpatients during lockdown were similar to those in previous years (Supplemental Table 5).

## Discussion

In a large secondary care hospital, the mean number of outpatients aged 50 years and older, attending the Fracture Clinic for a new suspected non-hip fragility fracture was significantly lower during lockdown, in 2020, compared with corresponding periods, across the previous five years. This decline was observed in both sexes and across age categories. Similar findings were found when restricting the analyses to only those outpatients with new confirmed fragility fractures. In contract, no change was observed in mean numbers of new inpatient admissions for acute hip fracture, during lockdown.

To our knowledge, our study is the first to report fragility fracture presentation among older adults during the COVID-19 pandemic. Additionally, our study shows that inpatient admissions for hip fracture did not change during this viral pandemic. A previous study reported that influenza illness may be associated with a modest 13% increase in risk of hospitalisation for hip fracture, among nursing homes residents [30].

Several possibilities could explain these findings. The reduction in attendance to the Fracture Clinic may reflect a true reduction in the incidence of new fragility fractures among older adults. The restriction of outdoor movement during lockdown may have led to fewer outdoor falls and subsequent fractures. Outdoor falls are a neglected public health problem [4–6]. At least half of the falls among community-dwelling older adults occur outdoors [4–7, 31–33]. Older adults with an active lifestyle who spend more time outdoors are at higher risk for outdoor falls and fractures [5, 7, 11]. Among middle-aged and older adults in Northern California, falls occurred outdoors more often than indoors and those who reported more leisure-time physical activity had a higher risk for outdoor falls [6]. Among older adults, mainly aged 70 years or older, in the MOBILIZE Boston study, almost half of the falls occurred outdoors and 9.2% of these resulted in serious injury, including fractures and non-fractures [4]. An early UK report emphasised that being housebound was associated with indoor falls, while walking for relaxation was associated with outdoor falls, among community-dwelling older adults [7]. In a multicentric US study, older community-dwelling women reported a wrist fracture more frequently outdoors than indoors, while hip fractures mainly occurred indoors [10].

In our study there was no reduction, during lockdown, of new inpatient admissions for acute hip fracture. In contrast to non-hip fractures, hip fractures mainly occur indoors [10]. In the large, international GLOW study, about two thirds of non-hip non-vertebral fracture occurred outdoors, while about half of hip fractures occurred indoors [8]. In other reports, an even higher proportion of hip fractures occurred indoors [9, 10, 34–36].

Another possibility to account for our findings may be that older adults decided not to attend the Fracture Clinic for fear of contracting COVID-19 in a hospital environment. However, given these fractures are extremely painful, this is unlikely. Furthermore, the lockdown legislation in the UK allowed the citizens to seek medical help, with no restriction [3]. In our view, it is unlikely that outpatients sought treatment outside of NHS hospitals as GP practices and private hospitals presented the same risk of transmission of SARS-CoV-2 and were frequently overwhelmed during the COVID-19 pandemic.

In our study, outpatients attending the Fracture Clinic were younger, during lockdown, compared with previous years. This may reflect better compliance to the lockdown rules among the oldest old adults, compared with those middle-aged. Moreover, a part of the middle-aged adults might be keyworkers in employment, who are allowed to move outdoors; in contrast, those aged 70 years and older might have been shielding at home.

## Strengths and limitations

Major strengths of our study are its novelty and its relevance. Further strengths are the prospective design, the setting in a large secondary care hospital, the inclusion of adults of both sexes and of a wide age range and that of all types of fragility fractures. A further strength of the design was to compare the mean number of attendances during lockdown to corresponding periods across the previous years; our choice took into account a vast literature on seasonality of many types of fractures, worldwide [14–20]. A few limitations have to be mentioned. Our study is based in a single centre. Moreover, we did not include patients who were admitted to hospital for medical reasons and concomitant acute fracture other than hip. Furthermore, data on the circumstances of the fracture were not collected. However, we could document a reduction in utilisation of the Fracture Clinic, during lockdown, which is relevant for healthcare planning. In view of our interest in healthcare utilisation, we performed our analyses in the total sample of outpatients attending the Fracture Clinic for suspected fragility fractures (including both confirmed and non-confirmed fragility fractures); our findings remained unchanged and robust, when restricting our analyses to those outpatients with confirmed fragility fracture. The findings of our study may be generalizable to other countries implementing lockdown.

## Conclusion

Our study showed a reduction in the mean number of older adults attending the Fracture Clinic, for non-hip fractures, during lockdown, but no reduction in inpatients admissions for acute hip fracture. This could result from the restriction of movements and the lack of opportunity for falling outdoors, during lockdown. Our report adds to previous literature on the heterogeneity of aetiology of fractures. Future research should explore the circumstances of falls and fractures, during lockdown. Our findings may be relevant to properly allocate limited healthcare resources in the context of a pandemic, in many countries that implement lockdown.

## Electronic supplementary material

ESM 1 (DOC 1744 kb)

## Data Availability

No additional data available.
