# Supplementary material for: The impact of lockdown during the COVID-19 pandemic on osteoporotic fragility fractures: an observational study
Source: Arch Osteoporos. 2020 Oct 7;15(1):156. doi: 10.1007/s11657-020-00825-1 (PMC7539555; doi:10.1007/s11657-020-00825-1)
Supplement: Supplementary file 1 — (DOC 1744 kb) [file 11657_2020_825_MOESM1_ESM.doc]

**The impact of lockdown during the COVID-19 pandemic on osteoporotic fragility fractures: an observational study**

**Giulia Ogliari, PhD^1^, Eleanor Lunt, MD, PhD candidate^1^, Terence Ong, MD^2^, Lindsey Marshall, nurse^3^, Opinder Sahota, professor^1^**

1. Department of Health Care for Older People, Queen’s Medical Centre, Nottingham University Hospitals NHS Trust, Nottingham, UK
2. Faculty of Medicine, University of Malaya, Kuala Lumpur, Malaysia
3. Department of Trauma & Orthopaedics, Queen’s Medical Centre, Nottingham University Hospitals NHS Trust, Nottingham, UK

**Journal title:** Osteoporosis International

**Corresponding Author:** Giulia Ogliari, Department of Health Care for Older People (HCOP), Queen’s Medical Centre, Nottingham University Hospitals NHS Trust, Derby Road, Nottingham, Nottinghamshire, NG7 2UH, UK, phone: +44 (0)115 924 9924 (extension 62067), e-mail: [Giulia.Ogliari1@nottingham.ac.uk](mailto:Giulia.Ogliari1@nottingham.ac.uk)

**Supplemental Figure 1. New outpatients to the Fracture Clinic in the first nineteen weeks of the year, across the years, by age category**

**
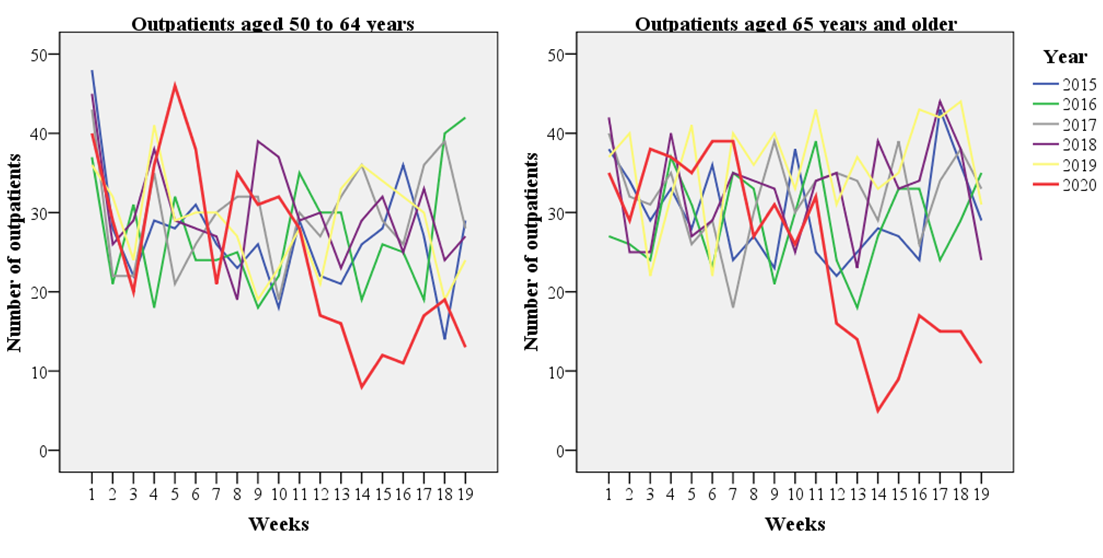
**

**Supplemental Figure 2. New outpatients to the Fracture Clinic in the first nineteen weeks of the year, across the years, by sex**

**
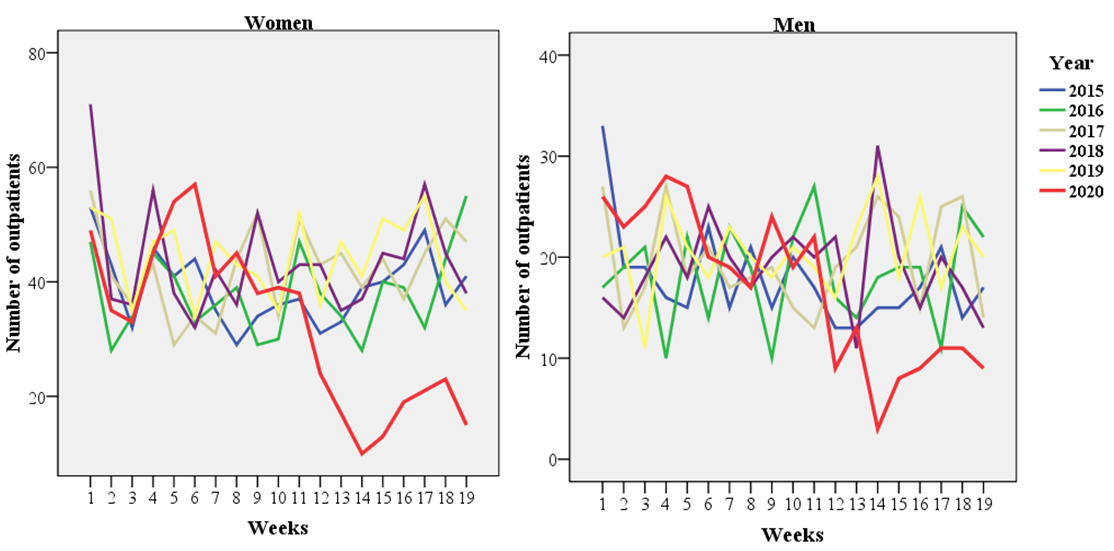
**

**Supplemental Figure 3. New outpatients to the Fracture Clinic in the first nineteen weeks of 2020, by age and sex categories**

**
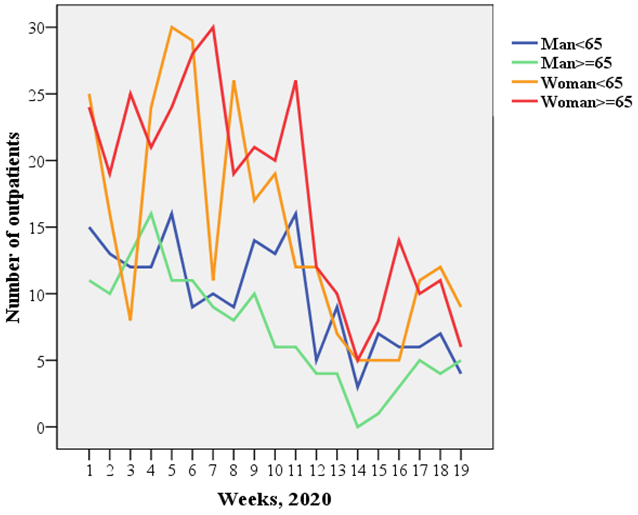
**

**Supplemental Figure 4. New outpatients to the Fracture Clinic in the first nineteen weeks of 2020, by fracture type**

**
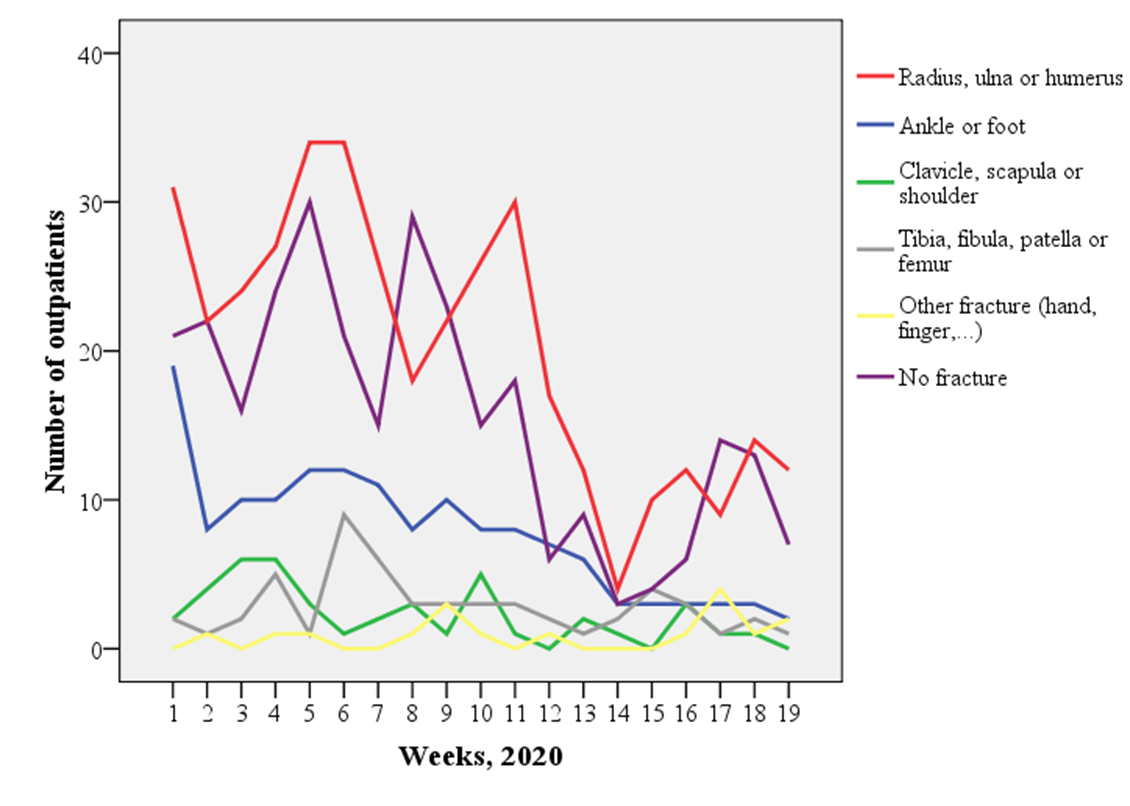
**

**Supplemental Table 1. Numbering of weeks, across the years, according to the UK calendar rules**

|  | 2015 | 2016 | 2017 | 2018 | 2019 | 2020 |
| --- | --- | --- | --- | --- | --- | --- |
| Week 1 | December 29, 2014 - January 4, 2015 | January 4, 2016 - January 10, 2016 | January 2, 2017 - January 8, 2017 | January 1, 2018 - January 7, 2018 | December 31, 2018 - January 6, 2019 | December 30, 2019 -January 5, 2020 |
| Week 2 | January 5, 2015 -January 11, 2015 | January 11, 2016 -January 17, 2016 | January 9, 2017 -January 15, 2017 | January 8, 2018 -January 14, 2018 | January 7, 2019 -January 13, 2019 | January 6, 2020 -January 12, 2020 |
| Week 3 | January 12, 2015 -January 18, 2015 | January 18, 2016 -January 24, 2016 | January 16, 2017 -January 22, 2017 | January 15, 2018 -January 21, 2018 | January 14, 2019 -January 20, 2019 | January 13, 2020 -January 19, 2020 |
| Week 4 | January 19, 2015 -January 25, 2015 | January 25, 2016 -January 31, 2016 | January 23, 2017 -January 29, 2017 | January 22, 2018 -January 28, 2018 | January 21, 2019 -January 27, 2019 | January 20, 2020 -January 26, 2020 |
| Week 5 | January 26, 2015 -February 1, 2015 | February 1, 2016 -February 7, 2016 | January 30, 2017 -February 5, 2017 | January 29, 2018 -February 4, 2018 | January 28, 2019 -February 3, 2019 | January 27, 2020 -February 2, 2020 |
| Week 6 | February 2, 2015 -February 8, 2015 | February 8, 2016 -February 14, 2016 | February 6, 2017 -February 12, 2017 | February 5, 2018 -February 11, 2018 | February 4, 2019 -February 10, 2019 | February 3, 2020 -February 9, 2020 |
| Week 7 | February 9, 2015 -February 15, 2015 | February 15, 2016 -February 21, 2016 | February 13, 2017 -February 19, 2017 | February 12, 2018 -February 18, 2018 | February 11, 2019 -February 17, 2019 | February 10, 2020 -February 16, 2020 |
| Week 8 | February 16, 2015 -February 22, 2015 | February 22, 2016 -February 28, 2016 | February 20, 2017 -February 26, 2017 | February 19, 2018 -February 25, 2018 | February 18, 2019 -February 24, 2019 | February 17, 2020 -February 23, 2020 |
| Week 9 | February 23, 2015 -March 1, 2015 | February 29, 2016 -March 6, 2016 | February 27, 2017 -March 5, 2017 | February 26, 2018 -March 4, 2018 | February 25, 2019 -March 3, 2019 | February 24, 2020 -March 1, 2020 |
| Week 10 | March 2, 2015 - March 8, 2015 | March 7, 2016 - March 13, 2016 | March 6, 2017 - March 12, 2017 | March 5, 2018 - March 11, 2018 | March 4, 2019 - March 10, 2019 | March 2, 2020 - March 8, 2020 |
| Week 11 | March 9, 2015 - March 15, 2015 | March 14, 2016 - March 20, 2016 | March 13, 2017 -March 19, 2017 | March 12, 2018 -March 18, 2018 | March 11, 2019 -March 17, 2019 | March 9, 2020 - March 15, 2020 |
| Week 12 | March 16, 2015 -March 22, 2015 | March 21, 2016 -March 27, 2016 | March 20, 2017 -March 26, 2017 | March 19, 2018 -March 25, 2018 | March 18, 2019 -March 24, 2019 | March 16, 2020 -March 22, 2020 |
| Week 13 | March 23, 2015 -March 29, 2015 | March 28, 2016 - April 3, 2016 | March 27, 2017 - April 2, 2017 | March 26, 2018 - April 1, 2018 | March 25, 2019 -March 31, 2019 | March 23, 2020 -March 29, 2020 |
| Week 14 | March 30, 2015 - April 5, 2015 | April 4, 2016 - April 10, 2016 | April 3, 2017 - April 9, 2017 | April 2, 2018 - April 8, 2018 | April 1, 2019 - April 7, 2019 | March 30, 2020 - April 5, 2020 |
| Week 15 | April 6, 2015 - April 12, 2015 | April 11, 2016 - April 17, 2016 | April 10, 2017 - April 16, 2017 | April 9, 2018 - April 15, 2018 | April 8, 2019 - April 14, 2019 | April 6, 2020 - April 12, 2020 |
| Week 16 | April 13, 2015 - April 19, 2015 | April 18, 2016 - April 24, 2016 | April 17, 2017 - April 23, 2017 | April 16, 2018 - April 22, 2018 | April 15, 2019 - April 21, 2019 | April 13, 2020 - April 19, 2020 |
| Week 17 | April 20, 2015 - April 26, 2015 | April 25, 2016 - May 1, 2016 | April 24, 2017 - April 30, 2017 | April 23, 2018 - April 29, 2018 | April 22, 2019 - April 28, 2019 | April 20, 2020 - April 26, 2020 |
| Week 18 | April 27, 2015 - May 3, 2015 | May 2, 2016 - May 8, 2016 | May 1, 2017 - May 7, 2017 | April 30, 2018 - May 6, 2018 | April 29, 2019 - May 5, 2019 | April 27, 2020 - May 3, 2020 |
| Week 19 | May 4, 2015 - May 10, 2015 | May 9, 2016 - May 15, 2016 | May 8, 2017 - May 14, 2017 | May 7, 2018 - May 13, 2018 | May 6, 2019 - May 12, 2019 | May 4, 2020 - May 10, 2020 |

**Supplemental Table 2. New outpatients attending the Fracture Clinic, over four (three) - week periods in the first nineteen weeks of the year, over the years 2015 to 2020**

|  | **2015** | **2016** | **2017** | **2018** | **2019** | **2020** | **P value** |
| --- | --- | --- | --- | --- | --- | --- | --- |
| **All outpatients, n**  Weeks 1^st^ to 12^th^ (prior to lockdown)  Weeks 13^th^ to 19^th^ (during lockdown) | 687  393 | 667  400 | 718  459 | 760  428 | 757  473 | 757  182 |  |
| **All outpatients, mean n (SD), per week** |  |  |  |  |  |  |  |
| **Weeks 1^st^ to 12^th^ (prior to lockdown)** | 57.3 (11.2) | 55.6 (9.1) | 59.8 (10.9) | 63.3 (10.9) | 63.1 (9.7) | 63.1 (12.6) | 0.338 |
| Weeks 1^st^ to 4^th^ | 65.3 (14.8) | 55.3 (6.9) | 65.0 (14.3) | 67.5 (17.7) | 66.0 (13.3) | 66.0 (9.3) | 0.805 |
| Weeks 5^th^ to 8^th^ | 55.8 (8.0) | 56.8 (6.8) | 53.0 (7.0) | 57.0 (3.7) | 63.8 (8.5) | 70.0 (10.6) | 0.057 |
| Weeks 9^th^ to 12^th^ | 50.8 (5.4) | 54.8 (14.5) | 61.5 (9.2) | 65.5 (4.5) | 59.5 (8.2) | 53.3 (13.6) | 0.326 |
| **Weeks 13^th^ to 19^th^ (during lockdown)** | 56.1 (7.7) | 57.1 (12.6) | 65.6 (7.8) | 61.1 (10.4) | 67.6 (6.6) | 26.0* (7.3) | < 0.001 |
| Weeks 13^th^ to 16^th^ | 53.8 (5.8) | 52.8 (6.7) | 62.8 (7.3) | 59.5 (9.7) | 70.8* (2.9) | 23.0** (7.7) | < 0.001 |
| Weeks 17^th^ to 19^th^ | 59.3 (10.1) | 63.0 (17.8) | 69.3 (8.0) | 69.3 (13.1) | 63.3 (8.5) | 30.0**^#^** (5.3) | 0.012 |

Trend analysis for mean number of new outpatients per week was performed with analysis of variance (ANOVA). Post-hoc Tukey’s test: * significant difference between the year 2020 and all the other years; **^**^**: significant difference between the year 2020 and all the other years and between the year 2019 and the years 2015 and 2016, respectively; **^#^** significant difference between the year 2020 and the years 2019, 2018, 2017 and 2016 (all p-values < 0.05). Abbreviation: SD: standard deviation.

**Supplemental Table 3. New outpatients with confirmed fractures attending the Fracture Clinic prior to lockdown and during lockdown in 2020 and in corresponding periods in the years 2015 to 2019 (n = 4,796)**

|  | **2015** | **2016** | **2017** | **2018** | **2019** | **2020** | **P value** |
| --- | --- | --- | --- | --- | --- | --- | --- |
| **Weeks 1^st^ to 12^th^ (prior to lockdown)** |  |  |  |  |  |  |  |
| All outpatients, n | 504 | 491 | 514 | 575 | 525 | 517 |  |
| All outpatients, mean n (SD), per week | 42.0 (10.2) | 40.9 (7.6) | 42.8 (11.7) | 47.9 (9.4) | 43.8 (7.8) | 43.1 (8.6) | 0.552 |
| Women, n | 363 | 349 | 380 | 432 | 391 | 385 |  |
| Women, mean n (SD), per week | 30.3 (6.1) | 29.1 (5.7) | 31.7 (9.1) | 36.0 (8.2) | 32.6 (6.1) | 32.1 (7.8) | 0.291 |
| Men, n | 141 | 142 | 134 | 143 | 134 | 132 |  |
| Men, mean n (SD), per week | 11.8 (5.1) | 11.8 (4.0) | 11.2 (3.5) | 11.9 (2.7) | 11.2 (3.2) | 11.0 (3.1) | 0.981 |
| Aged 50 to 64 years, n | 220 | 215 | 221 | 266 | 205 | 232 |  |
| Aged 50 to 64 years, mean n (SD), per week | 18.3 (6.4) | 17.9 (4.8) | 18.4 (6.5) | 22.2 (6.9) | 17.1 (3.7) | 19.3 (6.0) | 0.368 |
| Aged ≥ 65 years, n | 284 | 276 | 293 | 309 | 320 | 285 |  |
| Aged ≥ 65 years, mean n (SD), per week | 23.7 (5.2) | 23.0 (4.7) | 24.4 (6.6) | 25.8 (5.3) | 26.7 (5.4) | 23.8 (5.9) | 0.582 |
| **Weeks 13^th^ to 19^th^ (during lockdown)** |  |  |  |  |  |  |  |
| All outpatients, n | 290 | 281 | 335 | 315 | 323 | 126 |  |
| All outpatients, mean n (SD), per week | 41.4 (6.2) | 40.1 (7.8) | 47.9 (7.4) | 45.0 (7.0) | 46.1 (6.2) | 18.0 (4.1)* | < 0.001 |
| Women, n | 223 | 199 | 247 | 233 | 240 | 88 |  |
| Women, mean n (SD), per week | 31.9 (4.9) | 28.4 (5.6) | 35.3 (4.3) | 33.3 (6.2) | 34.3 (6.6) | 12.6 (2.2)* | < 0.001 |
| Men, n | 67 | 82 | 88 | 82 | 83 | 38 |  |
| Men, mean n (SD), per week | 9.6 (2.4) | 11.7 (3.5) | 12.6 (4.9) | 11.7 (4.5) | 11.9 (3.8) | 5.4 (2.1)# | 0.008 |
| Aged 50 to 64 years, n | 123 | 130 | 152 | 128 | 122 | 61 |  |
| Aged 50 to 64 years, mean n (SD), per week | 17.6 (3.9) | 18.6 (6.9) | 21.7 (3.9) | 18.3 (2.9) | 17.4 (3.3) | 8.7 (2.1)* | < 0.001 |
| Aged ≥ 65 years, n | 167 | 151 | 183 | 187 | 201 | 65 |  |
| Aged ≥ 65 years, mean n (SD), per week | 23.9 (5.6) | 21.6 (4.9) | 26.1 (4.3) | 26.7 (5.6) | 28.7 (5.5) | 9.3 (3.2)* | < 0.001 |

Trend analyses for mean numbers of outpatients per week were performed with analysis of variance (ANOVA). Post-hoc Tukey’s test: * significant difference between the year 2020 and all the other years; **^#^** significant difference between the year 2020 and the years 2016, 2017, 2018 and 2019 (all p-values < 0.05). Abbreviation: SD: standard deviation.

**Supplemental Table 4. New inpatients admitted for an acute hip fracture over four (three) - week periods in the first nineteen weeks of the year, over the years 2015 to 2020**

|  | **2015** | **2016** | **2017** | **2018** | **2019** | **2020** | **P value** |
| --- | --- | --- | --- | --- | --- | --- | --- |
| **All inpatients, n**  Weeks 1^st^ to 12^th^ (prior to lockdown)  Weeks 13^th^ to 19^th^ (during lockdown) | 211  94 | 162  106 | 151  107 | 183  118 | 204  106 | 197  113 |  |
| **All inpatients, mean n (SD), per week** |  |  |  |  |  |  |  |
| **Weeks 1^st^ to 12^th^ (prior to lockdown)** | 17.6 (5.4) | 13.5 (3.9) | 12.6 (2.7) | 15.3 (4.2) | 17.0 (6.4) | 16.4 (3.9) | 0.055 |
| Weeks 1^st^ to 4^th^ | 20.0 (7.4) | 16.3 (3.2) | 13.5 (3.7) | 16.0 (4.2) | 16.8 (8.7) | 15.8 (3.0) | 0.712 |
| Weeks 5^th^ to 8^th^ | 15.3 (4.3) | 11.8 (3.0) | 11.0 (1.6) | 17.8 (2.9) | 15.3 (3.9) | 16.5 (3.4) | 0.067 |
| Weeks 9^th^ to 12^th^ | 17.5 (4.4) | 12.5 (4.5) | 13.3 (2.2) | 12.0 (3.7) | 18.8 (6.8) | 17.0 (5.9) | 0.254 |
| **Weeks 13^th^ to 19^th^ (during lockdown)** | 13.4 (5.0) | 15.1 (3.5) | 15.3 (4.4) | 16.9 (3.6) | 15.1 (3.3) | 16.1 (5.6) | 0.776 |
| Weeks 13^th^ to 16^th^ | 13.0 (5.8) | 13.8 (2.9) | 13.0 (4.2) | 14.5 (1.7) | 14.8 (3.3) | 13.5 (1.0) | 0.934 |
| Weeks 17^th^ to 19^th^ | 14.0 (7.5) | 17.0 (4.0) | 18.3 (2.5) | 20.0 (3.0) | 15.7 (4.0) | 18.3 (8.5) | 0.781 |

Trend analysis for mean number of new inpatient admissions for acute hip fracture per week was performed with analysis of variance (ANOVA). No post-hoc Tukey’s test was statistically significant.

**Supplemental Table 5. Demographic characteristics of the new inpatients admitted for acute hip fracture during lockdown in 2020 and corresponding periods (13^th^ to 19^th^** week) across the years 2015 to 2019

|  | **2015**  **(n =94)** | **2016**  **(n =106)** | **2017**  **(n =107)** | **2018**  **(n =118)** | **2019**  **(n =106)** | **2020**  **(n =113)** | **P value** |
| --- | --- | --- | --- | --- | --- | --- | --- |
| Women, n (%) | 63 (67.0) | 66 (62.3) | 76 (71.0) | 69 (58.5) | 74 (69.8) | 77 (68.1) | 0.327 |
| Age (years), median (IQ) | 83 (79; 87) | 84 (78; 89) | 83 (77; 88) | 82 (75; 88) | 85 (77; 89) | 82 (75; 89) | 0.717 |
| Aged ≥ 85 years, n (%) | 37 (39.4) | 46 (43.4) | 39 (36.4) | 46 (39.0) | 56 (52.8) | 45 (39.8) | 0.438 |

P values are calculated using chi-square test for categorical variables and Kruskal-Wallis test for differences in median age. Abbreviations: n = number, IQ = interquartiles.
